# Prevalence and associated factors of viral hepatitis and transferrin elevations in 5036 patients admitted to the emergency room of a Swiss university hospital: cross-sectional study

**DOI:** 10.1186/1471-230X-7-5

**Published:** 2007-02-05

**Authors:** Stefan Russmann, Emmilia A Dowlatshahi, Gert Printzen, Susanne Habicht, Jürg Reichen, Heinz Zimmermann

**Affiliations:** 1 Department of Clinical Pharmacology, University of Berne, Berne, Switzerland; 2 Clinical Investigation Unit, University Hospital Berne, Berne, Switzerland; 3 Department of Epidemiology, Boston University School of Public Health, Boston, USA; 4 Institute of Clinical Chemistry, University Hospital Berne, Berne, Switzerland; 5 Emergency Department, University Hospital Berne, Berne, Switzerland; 6 Division of Hepatology, University Hospital Berne, Berne, Switzerland

## Abstract

**Background:**

The epidemiology of liver disease in patients admitted to emergency rooms is largely unknown. The current study aimed to measure the prevalence of viral hepatitis B and C infection and pathological laboratory values of liver disease in such a population, and to study factors associated with these measurements.

**Methods:**

Cross-sectional study in patients admitted to the emergency room of a university hospital. No formal exclusion criteria. Determination of anti-HBs, anti-HCV, transferrin saturation, alanine aminotransferase, and obtaining answers from a study-specific questionnaire.

**Results:**

The study included 5'036 patients, representing a 14.9% sample of the target population during the study period. Prevalence of anti-HBc and anti-HCV was 6.7% (95%CI 6.0% to 7.4%) and 2.7% (2.3% to 3.2%), respectively. Factors independently associated with positive anti-HBc were intravenous drug abuse (OR 18.3; 11.3 to 29.7), foreign country of birth (3.4; 2.6 to 4.4), non-white ethnicity (2.7; 1.9 to 3.8) and age ≥60 (2.0; 1.5 to 2.8). Positive anti-HCV was associated with intravenous drug abuse (78.9; 43.4 to 143.6), blood transfusion (1.7; 1.1 to 2.8) and abdominal pain (2.7; 1.5 to 4.8). 75% of all participants were not vaccinated against hepatitis B or did not know their vaccination status. Among anti-HCV positive patients only 49% knew about their infection and 51% reported regular alcohol consumption. Transferrin saturation was elevated in 3.3% and was associated with fatigue (prevalence ratio 1.9; 1.2 to 2.8).

**Conclusion:**

Emergency rooms should be considered as targets for public health programs that encourage vaccination, patient education and screening of high-risk patients for liver disease with subsequent referral for treatment if indicated.

## Background

Worldwide, about 350 million persons have chronic hepatitis B virus (HBV) infection, and about 125 million have been infected with hepatitis C virus (HCV), putting viral hepatitis B and C amongst the world's greatest infectious disease health problems [1-3]. Many patients are not aware of their chronic disease, and this is also true for another chronic liver disease, namely hereditary haemochromatosis, which is the most common autosomal recessive genetic disorder in Europe [4]. Viral hepatitis B and C, and haemochromatosis have in common that they may lead to liver cirrhosis and subsequently to liver failure and hepatocellular carcinoma, and that these complications can be prevented in most patients if diagnosis is made early and patients receive adequate treatment. Therefore these liver diseases are attractive candidates for public health measures aiming at prevention, early diagnosis and treatment. In this context not only information on the general population, but also on selected populations with a potentially higher risk is important. The setting of emergency rooms is particularly attractive for screening because patients can be directly educated about viral hepatitis, and treatment is readily available if necessary. A US based study determined the prevalence of HBV and HCV infection in an emergency room patient population in the early 1990s [5], but no comparable studies were done recently or in Europe. Likewise no previous studies have looked at the prevalence of elevated transferrin saturation (TFS) and its association with clinical symptoms of haemochromatosis and other liver diseases in such a population. We therefore aimed to study the epidemiology of these liver diseases in patients admitted to a university hospital emergency room.

## Methods

### Setting

The study was conducted in the emergency room (ER) of the University Hospital Berne where patients are admitted 24 hours per day, 365 days per year. The ER admits about 20'000 patients per year from urban Berne as well as from the surrounding rural areas. These include any medical, surgical and subspecialty emergencies, except paediatric, gynaecologic and obstetric emergencies, which are generally admitted to other specialised clinics. The study was approved and supervised by the regional ethics board (Kantonale Ethikkommission Bern).

### Subject recruitment and data collection

The a priori target was to include a representative sample of 5'000 patients from the population of all patients admitted to the ER. Patients were recruited between February 2003 and September 2004. There were no formal exclusion criteria, but any individual patient could only be included once. During an initial pilot phase of about two months we asked only a sample of patients admitted to the ER to participate in the study (increasing rate from 1 in 10 to 1 in 2 patients, selected by random sampling). Thereafter all patients were asked to participate in the study. Upon arrival and admission to the ER, patients were given a patient information sheet, an informed consent sheet, and a study-specific questionnaire, which was available in German, French, Italian and English (Table 1). Treating physicians and nurses provided additional information, answered any study-related questions and obtained a 10 ml venous blood sample. Demographic data were collected through the ER's electronic patient data system.

### Information on the source population

We obtained complete and reliable individual patient level information (age, sex, country of residence, health insurance, admission specialty, admission time and date) on all patients admitted to the ER during the study period from the hospital's electronic data system. This information was used to compare our study population with the source population, and to standardize prevalence to the source population.

### Laboratory analyses

Anti-HBc antibodies were determined with a diagnostic kit from Ortho-Clinical Diagnostics (Vitros Immunodiagnostic Products Anti-HBcAg Reagent Pack). Anti-HCV antibodies were determined using a second generation ELISA (Ortho-Clinical Diagnostics, Vitros Immunodiagnostic Products Anti-HCV Reagent Pack), measuring anti-HCV-C100-3, anti-C22c and anti-C33c. Serum alanine aminotransferase (ALT) activity, serum iron and total iron binding capacity were determined using the laboratory's routine methods. Transferrin saturation (TFS) was calculated as the ratio of serum iron and total iron binding capacity.

### Data analysis

Unadjusted prevalence was calculated directly, and 95% confidence intervals were based on a binomial distribution. For anti-HBc and anti-HCV antibodies prevalence was also adjusted by direct standardization to the source population's demographic and admission variables. Univariable (unadjusted) prevalence ratios (PR) were calculated for several independent variables (factors). When confounding by other variables was suspected adjusted relative prevalence rartios were calculated in a restricted population or as a pooled estimate after stratification over categories of potentially confounding variables according to the method of Mantel and Haenszel [6]. Thereafter multivariable logistic regression was used for the simultaneous modelling of independent associations with several variables. We used log-binomial regression as an alternative to logistic regression when log-binomial model fitting could be achieved, in order to estimate prevalence ratios instead of odds ratios [7]. All analyses were done with STATA Version 8.2 for MacOS X (Stata Corporation, College Station, Texas, USA).

## Results

### Demographic and admission data

During the study period a total of 33'703 patients were admitted to the ER; we collected complete data on 5'036 admitted patients, i.e. 14.9 % of the target population. Demographic and admission data of the study and source population are presented in Table 2. As shown, the two populations had overall similar distributions for the available variables, but study participants were more likely to be admitted during daytime and during the summer months, which is most likely attributable to logistic factors of the study.

### Questionnaires

**Figure 1:**
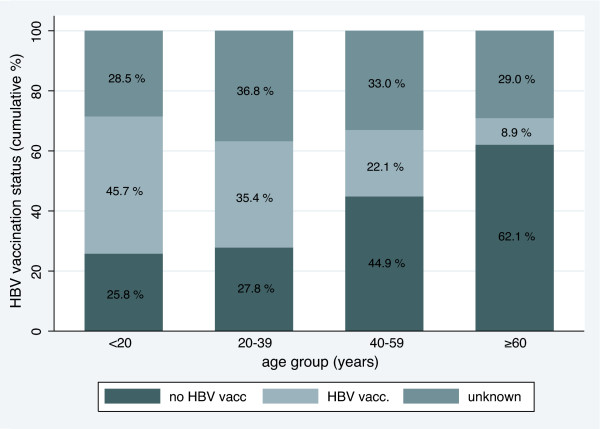
Hepatitis B vaccination status according to patient questionnaires over 4 age categories. As shown, knowledge about vaccination status does not change in relation to age, but the proportion of patients without HBV vaccination increases above the age of 40.

Completed questionnaires were available for all included patients. Some patients did not answer all questions, but each question was answered by at least 97%. Particularly those questions that addressed history of HBV and HCV infection revealed interesting results: 42% of all included patients reported that they had not been vaccinated against HBV, and another 33% did not know whether they had been vaccinated. Not surprisingly the proportion of HBV-vaccinated patients decreased with higher age (Figure 1). 86% of anti-HBc positive patients were not aware of their previous HBV infection, and 74% mentioned no history of jaundice. Only 57% of patients who reported a previous HBV infection were anti-HBc positive. Specificity of a positive history of HCV infection obtained through the study questionnaires was also relatively low (73% of those who reported a previous HCV infection were anti-HCV positive), but the proportion of patients who knew about their previous infection was about three times higher for HCV than for HBV (49% vs. 14%). Information on drinking habits obtained from the questionnaires showed that 10% of men vs. 2.3% of women reported consumption of more than 10 alcoholic drinks per week. Among HCV positive patients, 34% had between 1 and 10, and 16% more than 10 alcoholic drinks per week. HCV positive patients therefore had a higher prevalence of having >10 drinks per week (PR 2.5, 95% CI 1.7 to 3.7), and it was not lower in patients who were aware of their HCV infection (PR 2.7). Importantly, patients who knew about their HCV infection did not drink less alcohol. Other results from the questionnaires are presented in Table 3 along with their associations with viral hepatitis serology.

### Alanine aminotransferase (ALT)

ALT was determined in all but five patients of the study population. Results are presented in Table 4. As shown, women had about half the prevalence of elevated ALT compared to men. There were no pronounced variations over age categories. In search of the most common causes of *severe *ALT elevations, we also reviewed the ER physicians' reports on all 130 studied patients with an ALT ≥100 U/l, and derived the most likely cause of their elevated ALT. The most common causes were alcohol consumption (n = 18), infection (n = 17), obstructive cholestasis (n = 16), pancreatitis (n = 10), drug-induced (n = 10), abdominal trauma (n = 9) and HCV infection (n = 8). In 28 patients the reports did not provide any information that readily explained their elevated ALT.

### Prevalence of anti-HBc and anti-HCV and associated factors

Out of 5'036 included patients 336 were anti-HBc, and 135 were anti-HCV positive. 46 patients were positive for both anti-HBc and anti-HCV. There were 15 borderline test results for anti-HBc antibodies and 6 for anti-HCV antibodies. All other patients had negative results. Of the 135 anti-HCV positive patients, 21 were later seen in our hepatological outpatient clinic, where anti-HCV was determined with recombinant immunoblot assays (RIBA), and HCV RNA was measured using polymerase chain reaction. Nineteen tested both, RIBA and RNA positive, one RIBA positive and RNA negative, and one RIBA and RNA negative. In this sample, the predictive value positive regarding anti-HCV was therefore 20/21 or 95.2% after confirmatory RIBA. Table 5 shows the resulting observed prevalences of viral hepatitis serology along with estimates that were standardised to the demographic and admission variables of the source population. Anti-HBc prevalence was substantially higher in patients with a foreign country of birth, i.e. 4.5% for patients born in Switzerland vs. 14.4% for those born in any other country, 15.9% for Mediterranean countries, and 20.1% amongst 102 patients born in Italy. Table 3 presents viral serology results in correlation to questionnaire answers, demographic and admission characteristics and laboratory results, and provides univariable prevalence ratios for positive viral serology. This unadjusted analysis suggested an association between positive anti-HCV antibodies and tattoos (PR = 3.4). However, in a stratified Mantel-Haenszel analysis that controlled for IVDA as a confounding factor the adjusted RR was only 1.3 (95% CI 0.9 to 1.7), and also the alternative approaches of an analysis in a restricted study population of patients without IVDA and a multivariable analysis (Table 6) provided similar results. After data exploration with stratified and restricted analyses we selected clinically relevant factors that were associated with positive viral hepatitis serology in the univariable analysis and those that were considered as potential confounders as independent variables in a logistic regression model. The resulting adjusted odds ratios for viral serology are presented in Table 6.

### Transferrin saturation (TFS) and associated factors

TFS was available for all but one patient in the study population. 221 patients had a TFS >45% (4.4%; 95% confidence interval 3.8 to 5.0), 145 >50% (2.9%, 2.4 to 3.4), and 97 >55% (1.9%, 1.6 to 2.3). Applying sex-specific limits (>45% in women and >50% in men) prevalence of elevated TFS was 3.3 % (2.8 to 3.8). We observed no marked age-dependent differences. Univariable analysis indicated associations of elevated TFS with elevated ALT (PR 1.5; 1.0 to 2.1), high alcohol consumption (PR 2.4; 1.6 to 3.6), and fatigue (PR 1.9; 1.4 to 2.7), but not with positive anti-HCV (PR 1.1; 0.5 to 2.7). Restriction of the analysis to patients without high alcohol intake, positive anti-HCV or IVDA (remaining n = 4'457) did not substantially change the association of elevated TFS with fatigue or ALT. Using the same restrictions we also fitted a log-binomial regression model with elevated TFS as the dependent variable and abdominal pain, fatigue, myalgia and arthralgia (possible symptoms of haemochromatosis) as independent variables. This model showed a virtually identical association of elevated TFS with fatigue (PR 1.9; 1.2 to 2.8), but no significant associations with abdominal pain, myalgia or arthralgia. Exclusion of non-white subjects did not substantially change these associations.

## Discussion

The current study presents the first data on prevalence and associated factors of liver diseases in a large sample of patients admitted to the emergency room of a European university hospital. The prevalence of anti-HBc and anti-HCV was 6.7% and 2.7%, respectively. Factors independently associated with positive anti-HBc were IVDA, foreign country of birth, non-white ethnicity and high age. Positive anti-HCV was associated with IVDA, blood transfusion and chronic abdominal pain. Patients demonstrated poor awareness and knowledge of liver disease. Transferrin saturation was elevated in 3.3% and was associated with chronic fatigue.

The obtained data from a large sample of patients admitted to the ER were essentially complete, plausible, and generally in accordance with previously available information, supporting the overall validity of our study. The current study also had important limitations: first, we studied only 14.9% of our target population, and the possibility of a recruitment bias has to be addressed. Therefore, we obtained demographic and admission characteristics on all patients admitted to the ER during the study period, and a comparison with this information demonstrated that our study population can be considered as a reasonably representative sample of the target population with regard to these variables, and allowed us to standardise prevalence estimates. Second, we were not able to perform additional laboratory tests of interest including HBsAg, anti-HBs, and confirmatory secondary testing in all anti-HCV positive patients. However, like in other studies, the primary aim of our study was to determine past infection with HBV and HCV. Also, further confirmatory testing was done in a small sample of anti-HCV positive patients, and the observed predictive value positive of 95% suggests that among the 135 anti-HCV positive patients 129 had a previous HCV infection. Assuming a 94% sensitivity of the study's enzyme immunoassay [8], about 137 patients in the tested population would then have had a previous HCV infection, which is very similar to the number without these corrections. And although HCV-RNA was not determined in all patients, one can expect that approximately 90% of infected patients had a chronic infection [9]. Third, we were not able to do genotyping for mutations associated with haemochromatosis. Finally, the observed associations are descriptive, and do not allow causal inferences.

Several studies from different countries have reported prevalence estimates of viral hepatitis in selected or more general populations as e.g. in the Italian Dionysos study [10]. However the only previous study from a developed country that determined the prevalence of viral hepatitis in an emergency room population was conducted in Baltimore, USA in the early 1990s, i.e. before the introduction of widespread HBV vaccination programs [5]. There, the prevalence was 5% for HBsAg, indicating active and not previous HBV infection, and 18% for anti-HCV. In the general Swiss population an estimated 3% have been infected with HBV [11,12], and 0.75% with HCV [13]. Our results therefore indicate that the prevalence of previous infection is about twice as high for HBV and three times higher for HCV compared to the general population. Possible explanations for the higher prevalence are medical conditions associated with viral hepatitis that lead to ER admission and overrepresentation of high-risk groups such as intravenous drug users, patients not enrolled in regular health plans and a primarily urban population. Amongst intravenous drug users, anti-HBc prevalence was only 11.6% compared to 60–80% amongst Swiss IVDA cohorts in the 1980s and 20% in the early 1990s [14,15]. The higher prevalence of previous HBV infection in immigrants is in accordance with frequent perinatal transmission in countries with high endemicity, and with results from a previous Swiss study in pregnant women [12]. 60% of intravenous drug users were anti-HCV positive, which is similar to the results of two Swiss studies published in 1990 and 2000 in IVDA cohorts [16,17].

The decreasing prevalence of HBV infections in intravenous drug users demonstrates the success of HBV vaccination programs that target this high-risk group, and immunization of all adolescents (implemented in Switzerland in 1998), is expected to be eventually highly efficacious in reducing HBV infections in the general population. However it will require decades until these measures will reduce the population burden of HBV infection, and adults at high risk including immigrants are difficult to reach through national vaccination programs and public health campaigns. Particularly this high-risk subpopulation is well represented in ER departments. Further, 74% of the anti-HCV positive patients admitted to the ER were less than 60 years old, which is of particular relevance since treatment with pegylated interferon and ribavirin should be instituted in patients at risk for late complications at a young age. Based on a chronic course in approximately 10% of anti-HBc and 70–80% of anti-HCV positive patients, about 130 patients with chronic HBV infection and 350–400 patients with chronic HCV infection are seen in the studied ER population during one year. However 42% had not been vaccinated against HBV, 33% did not know whether they had been vaccinated, only less than half of all anti-HCV positive patients knew about their infection, and half of all anti-HCV positive patients reported regular alcohol consumption, which is strongly discouraged as it promotes HCV-related fibrogenesis [18]. Therefore our results indicate that intense efforts to diagnose viral hepatitis and educate patients in ER have the potential to prevent new infections and treat complications.

Elevated TFS is an important marker of disease in C282Y homozygote patients, who account for about 90% of all clinical cases of haemochromatosis [19,20]. Although the prevalence of this mutation is known to be about 0.4 to 0.5% in Caucasian populations [4,21], until recently there has been few data on the penetrance of C282Y in the general population, which is critically important for the controversial discussion on the utility of population screening [22]. In a large international study Adams et al. reported a prevalence of 0.44% for C282Y homozygotes in a white primary care population, and amongst those TFS was >50% in 84% of men and >45% in 73% of women [21]. Based on this data we would expect 20 C282Y homozygous amongst 4'616 white patients (60% men) in our study population, and 16 of them with elevated TFS as defined above. We identified 151 patients with such an elevated TFS amongst 4'616 white patients (3.3%), which allows us to estimate a specificity of 97% and a positive predictive value of 11% regarding the identification of C282Y homozygote patients with elevated TFS in our study population. This implies that 89% of patients with an elevated TFS in white patients of our study population were not C282Y homozygote and therefore limits the power and specificity of our results with regard to haemochromatosis. Other studies in primary care populations were able to identify patients with C282Y homozygosity through genetic screening and reported an association with liver diseases. However, they also failed to observe an association between C282Y homozygosity with elevated TFS and the rather non-specific symptoms of haemochromatosis that are highly prevalent in such a population [21,23,24]. Further, our study had the advantage that patients with high alcohol consumption and HCV infection could be excluded in a subanalysis, and the higher prevalence of fatigue in patients with elevated TFS even within this restricted population may indicate the presence of other chronic liver diseases including haemochromatosis and non-alcoholic steatohepatitis.

Recent data suggest that even amongst C282Y homozygotes with elevated TFS only a minority will develop frank clinical haemochromatosis, and consequently no clear indication for population-based screening for haemochromatosis has been established [22,24]. Nevertheless, increased TFS is a useful sign of chronic liver disease including haemochromatosis and non-alcoholic steatohepatitis, and our results confirm that chronic fatigue is a possible symptom of increased TFS and chronic liver disease.

## Conclusion

We conclude that emergency rooms should be considered as targets for public health programs that encourage vaccination, patient education and screening of high-risk patients for liver disease with subsequent referral for treatment if indicated. Future studies may compare our results with the epidemiology of liver disease in emergency rooms in other hospitals and countries, and evaluate the effect of preventive measures.

## Authors' contributions

SR lead the study coordination and the work of the Clinical Investigation Unit, contributed to the study design, undertook all statistical data analyses, and wrote the first draft of the manuscript. EAD contributed to the data collection, organized the raw data files and undertook the initial descriptive data analyses. HZ and JR initiated and designed the study, revised the manuscript and obtained the funding. HZ lead the contributions of the emergency room staff. GP was responsible for all laboratory sample analyses. SH contributed to the initial study design and planning. All authors read and approved the final manuscript.

## Pre-publication history

The pre-publication history for this paper can be accessed here:



## Figures and Tables

**Table 1:** Study-specific patient questionnaire

**Questions**	**Answer options**
Have you been vaccinated against hepatitis B ?	Yes/No/I don't know
Do you have any known hepatitis B infection ?	Yes/No
Do you have any known hepatitis C infection ?	Yes/No
Have you ever had a yellowing of the skin (known as jaundice) ?	Yes/No
Have you ever had a blood transfusion ?	Yes/No, if yes until/after 1990 ?
Do you have a tattoo ?	Yes/No
Do you have any piercing ?	Yes/No
Do you drink alcohol ?	Never/1–10/>10 glasses/week
Have you ever taken illegal intravenous drugs ?	Yes/No
Do you live with a partner in a steady relationship ?	Yes/No
Do you work in the public health services with patient contact ?	Yes/No
Have you been suffering from stomach aches without clear cause ?	Yes/No
Have you been suffering from tiredness without clear cause ?	Yes/No
Have you been suffering from muscle pains without clear cause ?	Yes/No
Have you been suffering from joint pains without clear cause ?	Yes/No
What is your ethnic origin ?	White/Black/Asiatic/other, mixed
Were you born in Switzerland ?	Yes/No, if no specifiy:
Do you permanently reside in Switzerland ?	Yes/No, if no specifiy:

**Table 2:** Demographic and admission data of the study and source population

		***Study population***		***All ER admissions***^1^	
**Variable**	**Strata**	**n**	**%**	**n**	**%**

All patients		5'036	100	33'703	100
					
Sex	Male	3'018	59.9	19'397	57.6
	Female	2'018	40.1	14'299	42.4
	Unknown	-	-	7	0.02
					
Age (years)	< 20	199	4.0	2'380	7.0
	20–39	1'890	37.5	12'936	38.4
	40–59	1'610	32.0	9'460	28.1
	≥ 60	1'337	26.5	8'927	26.5
					
Admission specialty	Surgery	2'632	52.3	19'730	58.5
	Medicine	2'336	46.4	13'973	41.5
	Unknown	68	1.3	-^2^	-
					
Admission season	Nov-Apr	1'693	33.6	14'344	42.6
	May-Oct	3'343	66.4	19'359	57.4
					
Admission time	Day (7 am – 7 pm)	4'112	81.7	22'917	68.0
	Night (7 pm – 7 am)	856	17.0	10'786	32.0
	Unknown	68	1.3	-^2^	-
					
Country of residence	Switzerland	4'934	98.0	32'853	97.5
	Other	102	2.0	850	2.5
					
Health insurance	Basic	3'964	78.7	27'595	81.9
	Semi-private	685	13.6	3'650	10.8
	Private	165	3.3	970	2.9
	Unknown	222	4.4	1'488	4.4

^1^All admissions to the emergency room of the University Hospital Berne between 26 February 2003 and 20 September 2004 (data obtained via the hospital's electronic patient data system)

^2^There were 68 patients who entered the ER and were included in the study, but where the hospital's data system did not have an entry and therefore no information on specialty and time of admission. These are not included in the numbers provided for all admissions.

**Table 3:** Univariable prevalence ratios (PR) for positive viral hepatitis serology

	**Negative hepatitis serology (n = 4'592)**		**Anti-HBc positive (n = 336)**				**Anti-HCV positive (n = 135)**			
**Factor**	**n**	**(%)**^1^	**n**	**(%)**^1^	**PR**^2^	**(95%CI)**	**n**	**(%)**^1^	**PR**^2^	**(95%CI)**

*Demographics*										
Female	1'859	(40.5)	117	(34.8)	0.8	(0.6 to 1.0)	44	(33.1)	0.7	(0.5 to 1.0)
Age										
≤39	1'957	(42.6)	129	(38.4)	1.0	(reference)	59	(43.7)	1.0	(reference)
40–59	1'440	(31.4)	118	(35.1)	1.2	(1.0 to 1.5)	45	(33.3)	1.1	(0.8 to 1.6)
≥60	1'195	(26.0)	89	(26.5)	1.1	(0.9 to 1.4)	31	(23.0)	1.0	(0.7 to 1.5)
										
*Admission*										
Night admission (7 pm–7 am)	625	(15.6)	39	(13.9)	0.9	(0.6 to 1.2)	15	(14.0)	0.9	(0.5 to 1.5)
Nov-April admission	1'557	(33.9)	106	(31.5)	0.9	(0.7 to 1.1)	44	(32.6)	0.9	(0.7 to 1.3)
Medical admission	1'855	(46.3)	153	(54.4)	1.4	(1.1 to 1.7)	55	(40.7)	1.2	(0.8 to 1.7)
(Semi-)private insurance	800	(18.2)	32	(9.9)	0.5	(0.4 to 0.7)	15	(11.8)	0.6	(0.4 to 1.0)
										
*Laboratory results*										
Elevated ALT	835	(18.2)	91	(27.1)	1.6	(1.3 to 2.0)	56	(41.5)	3.1	(2.2 to 4.3)
Transferrin saturation >45%	200	(4.4)	14	(4.2)	1.0	(0.6 to 1.6)	9	(6.7)	1.5	(0.8 to 3.0)
										
*Questionnaire*										
History of jaundice	428	(9.5)	88	(26.9)	3.1	(2.5 to 3.9)	39	(29.5)	3.8	(2.6 to 5.4)
Blood transfusion (any time)	945	(21.2)	71	(22.0)	1.0	(0.8 to 1.3)	38	(30.9)	1.6	(1.1 to 2.4)
Blood transfusion before 1990	453	(10.2)	30	(9.3)	0.9	(0.6 to 1.3)	15	(12.2)	1.2	(0.7 to 2.1)
Tattoo	437	(9.6)	43	(12.9)	1.4	(1.0 to 1.9)	37	(27.4)	3.4	(2.3 to 4.9)
Piercing	486	(10.7)	25	(7.5)	0.7	(0.5 to 1.0)	22	(16.4)	1.6	(1.0 to 2.5)
>10 units alcohol per week	294	(6.5)	37	(11.2)	1.7	(1.3 to 2.4)	22	(16.4)	2.7	(1.7 to 4.2)
Intravenous drug abuse	30	(0.7)	39	(11.7)	9.3	(7.4 to 11.8)	55	(40.7)	37.4	(28.6 to 48.8)
Steady relationship	3'221	(70.9)	229	(70.0)	0.9	(0.7 to 1.2)	72	(53.7)	0.5	(0.4 to 0.7)
Professional patient contact	618	(13.6)	41	(12.4)	0.9	(0.7 to 1.2)	14	(10.4)	0.7	(0.4 to 1.3)
Chronic abdominal pain	373	(8.2)	47	(14.2)	1.8	(1.3 to 2.4)	30	(22.7)	3.1	(2.1 to 4.6)
Chronic fatigue	817	(17.9)	84	(25.2)	1.5	(1.2 to 1.9)	45	(33.6)	2.2	(1.6 to 3.2)
Chronic myalgia	467	(10.2)	42	(12.6)	1.2	(0.9 to 1.7)	21	(15.8)	1.6	(1.0 to 2.6)
Chronic arthralgia	568	(12.5)	57	(17.3)	1.4	(1.1 to 1.9)	27	(20.6)	1.8	(1.2 to 2.7)
Ethnicity other than white	244	(5.4)	65	(19.8)	3.6	(2.8 to 4.6)	10	(7.5)	1.4	(0.7 to 2.6)
Not born in Switzerland	926	(20.3)	160	(47.8)	3.2	(2.6 to 3.9)	30	(22.2)	1.1	(0.8 to 1.7)
Not a Swiss resident	92	(2.0)	7	(2.1)	1.0	(0.5 to 2.1)	2	(1.5)	0.7	(0.2 to 2.9)

^1^If no information was available for individual patients for a certain factor (e.g. admission time), % is calculated as the proportion of all patients with available information.

^2^Unadjusted prevalence ratio of positive viral serology for present vs. absent factor (e.g. first row: prevalence ratio of positive anti-HBc in females vs. males), except for age where age ≤39 is defined as the reference category.

**Table 4:** Sex-specific distribution of ALT expressed as upper limits of normal (ULN) over 4 categories

**Men**	**n**	**%**
≤1× ULN	2'290	75.9
> 1 – 3× ULN	626	20.7
> 3 – 10× ULN	83	2.8
> 10× ULN	19	0.6
		
**Women**	**n**	**%**

≤1× ULN	1'783	88.3
> 1 – 3× ULN	199	9.9
> 3 – 10× ULN	28	1.4
> 10x ULN	8	0.4

**Table 5:** Observed and standardised prevalences of positive anti-HBc and anti-HCV antibodies in the study population of 5'036 patients

**Prevalence**	**Anti-HBc + % (95% CI)**	**Anti-HCV + % (95% CI)**
Observed	6.7 (6.0 to 7.4)	2.7 (2.3 to 3.2)
Standardized to age^1^	6.5 (5.9 to7.2)	2.6 (2.2 to 3.1)
Standardized to admission variables^1^	6.1 (5.3 to 6.9)	2.4 (1.9 to 2.8)

^1^Direct standardisation to distribution of age or admission variables (season, daytime and specialty) of the source population, i.e. all 33'703 patients admitted to the emergency room during the study period.

**Table 6:** Adjusted odds ratios for variables associated with positive viral hepatitis serology

**Variables**^1^	**Odds ratio**	**(95%CI)**
	***Anti-HBc positive***	

Female	0.8	(0.7 to 1.1)
Age		
≤39	1.0	(reference)
40–59	1.5	(1.1 to 2.0)
≥60	2.0	(1.5 to 2.8)
Intravenous drug abuse	18.3	(11.3 to 29.7)
Ethnicity not white	2.7	(1.9 to 3.8)
Not born in Switzerland	3.4	(2.6 to 4.4)
>10 units alcohol per week	1.5	(1.0 to 2.2)
		
	***Anti-HCV positive***	

Female	0.8	(0.5 to 1.2)
Age		
≤39	1.0	(reference)
40–59	1.7	(1.0 to 2.9)
≥60	1.7	(0.9 to 3.1)
Blood transfusion	1.7	(1.1 to 2.8)
Tattoo	1.7	(0.9 to 3.2)
Piercing	1.1	(0.5 to 2.2)
Intravenous drug abuse	78.9	(43.4 to 143.6)
>10 units alcohol per week	1.1	(0.5 to 2.2)
Steady relationship	0.8	(0.5 to 1.3)
Chronic abdominal pain	2.7	(1.5 to 4.8)
Chronic fatigue	1.2	(0.7 to 2.0)
Chronic arthralgia	1.2	(0.7 to 2.2)

^1^Multivariable logistic regression models. First column presents all independent variables that were included in the two models with HBV or HCV serology as the dependent variable.
